# Research priorities for antimicrobial stewardship nurses in a middle-income country: a nominal group technique study

**DOI:** 10.1186/s12912-024-02504-9

**Published:** 2024-12-02

**Authors:** Viviane Cristina de Lima Gusmão, Lígia Maria Abraão, Adriana Maria da Silva Felix, Caroline Lopes Ciofi-Silva, Molly Courtenay, Valerie Ness, Enrique Castro-Sanchez, Rosely Moralez de Figueiredo, Maria Clara Padoveze, Workshop Participants Group, Monik Gomes do Nascimento Lousada, Claudia Silva Marinho, Eliana Auxiliadora Magalhães Costa, Nayara Carvalho Oliveira, Waldélia Monteiro, Beatriz Murata Murakami, Andreza Manhezi, José Rodrigues do Carmo Filho, Zilah Cândida Pereira das Neves, Viviane Gonçalves Sena, Glaucia Ribeiro Goncalves, Ieda Pontes da Cruz, Fernando Augusto Pinheiro, Camila Piuco Preve, Eliane Carlosso Krummenauer, Renata Neto Pires, Amanda Luiz Pires Maciel, Ana Claudia Cascardo, Daiane Patrícia Cais, James Francisco Pedro dos Santos, Lilian Farah, Maria Fernanda Zorzi Gatti, Meire Cristina Novelli e Castro, Mônica Taminato, Thatiara Cardoso da Silva, Tiago Cristiano de Lima

**Affiliations:** 1 Department of Collective Health Nursing, School of Nursing, University of São Paulo, São Paulo, Brazil; 2 Nursing School, University of Campinas, Campinas, Brazil; 3 School of Health Sciences, Cardiff University, Cardiff, UK; 4 Department of Nursing and Community Health, Glasgow Caledonian University, Glasgow, UK; 5 Brunel University London, London, UK; 6 Global Health Research Group, University of the Balearic Islands, Palma, Spain; 7 Nursing Department, Federal University of São Carlos, São Carlos, Brazil

**Keywords:** Nursing, Nursing research, Antimicrobial stewardship, Antimicrobial resistance, Nominal group technique, Group processes

## Abstract

**Background:**

Antimicrobial stewardship programs (ASPs) have become important strategies for addressing antimicrobial resistance (AMR). Despite the increasing number of international publications identifying the important roles played by nurses as part of ASPs in low- and middle-income countries, this topic is yet poorly researched. This study aimed to identify priority research gaps in the Brazilian context concerning nurses’ performance in ASPs from the perspective of nursing professionals and explore the main themes among the ideas generated by these nurses.

**Methods:**

This qualitative study used the modified Nominal Group Technique (mNGT) during a three-day online workshop. Content analysis was performed on the basis of the ideas proposed by the participants after the clarification stage.

**Results:**

The participants suggested 68 ideas in the first phase. After the idea’s clarification phase, 45 ideas were included in the voting rounds. The ideas prioritized by participants voting addressed (i) attributions and competencies of nurses in the ASP; (ii) planning and implementation of ASP nurses’ activities; and (iii) use of information and communication technologies to assist nurses. The content analysis highlighted nine main themes in the initial ideas.

**Conclusions:**

The study identified significant gaps in research related to nurses’ roles in ASPs in the Brazilian context. These findings suggest that further investigation into nurses’ competences, the implementation of their roles, and the application of digital tools are priority subjects of future research to improve nurses’ participation in ASPs. These themes should be further studied in the Brazilian context but may be applicable to other similar socioeconomic contexts.

**Supplementary Information:**

The online version contains supplementary material available at 10.1186/s12912-024-02504-9.

## Background

Antimicrobial resistance (AMR) is the ability of a microorganism to overcome the effects of antimicrobial agents, resulting in their ineffectiveness [1]. AMR has emerged as a major global public health challenge with sociopolitical consequences, overwhelming health systems and threatening the sustainability of contemporary society [2–4]. Antimicrobials have contributed to advances in the treatment and prophylaxis of infections, allowing therapeutic procedures such as transplants and complex surgeries. However, the inappropriate use of antimicrobials, fostered by poverty and a lack of hygiene and sanitation, has encouraged the development of AMR worldwide [5–8].

The Global Action Plan on AMR adopted by the World Health Assembly in 2015 aimed to identify, manage, and mitigate the risks associated with AMR [9]. A strategic objective includes optimizing the use of antimicrobials through antimicrobial stewardship programs (ASPs), which are multimodal interventions requiring the interdisciplinary and collaborative engagement of health care professionals [10]. However, most ASP guidelines typically recognize the contributions of physicians, pharmacists, and microbiologists but rarely include nurses [11–13]. In many situations, nursing engagement in ASPs is most often seen through the lens of infection prevention and control (IPC) [14–16]. Nonetheless, nurses perform other pivotal actions towards optimal management of antibiotics and infections within their scope of practice, including central roles as communicators and coordinators of care between health professionals, patients, and their families [11, 17, 18].

Nursing holds a significant position among healthcare professionals because of its major contribution to patient care [11, 18, 19]. The evidence suggests that nurses may contribute by assessing and monitoring patients’ clinical status, participating in diagnostic and antimicrobial treatment management, and educating patients and families [18, 20, 21]. However, in Brazil, there are still research gaps related to nurses’ role in ASPs, which need to be identified and prioritized for future investigations to strengthen the contribution of nurses in these programs [20, 22]. Addressing these gaps would be particularly valuable for low- and middle-income countries, where the contribution of nursing in combating AMR is unknown or underestimated [23].

## Methods

This study aimed at identifying priority research gaps in the Brazilian context concerning nurses’ performance in ASPs from the perspective of nursing professionals and to explore the main themes among the ideas generated by these nurses.

### Study design

The study was a qualitative study using the Nominal Group Technique (NGT) modified to adapt to a virtual scenario (mNGT). The NGT is a structured method for the development of consensus, using a focus group modality, in which the contribution of the participants is obtained through the presentation of a predetermined guiding question during the discussion. Answers are obtained from the participants through the presentation of a question on the topic addressed [24]. It is formally structured in four stages: generation of ideas, presentation of ideas, clarifications and voting [25]. Answers are obtained from the participants through the presentation of a question on the topic addressed. NGT allows participants to identify, evaluate, and rank important aspects of a specific topic or problem [26]. In health research, both the NGT and the Delphi technique serve as valuable methods for gathering and synthesizing expert opinions [27]. Compared with the Delphi technique, NGT has a lower drop-out rate throughout the process [28]. While NGT involves structured face-to-face group discussions, the Delphi technique relies on iterative rounds of anonymous surveys among geographically dispersed experts [28]. Additionally, NGT typically results in quicker consensus due to real-time interaction, whereas the Delphi technique may require multiple rounds of feedback and analysis, potentially extending the timeline. Research in health science illustrates the importance of the NGT as a powerful tool for gathering insights and perspectives from diverse stakeholders [25, 26, 29]. NGT has also been applied to identify research priorities in various stages of research, from problem identification to solution generation [30, 31]. The decision to employ the mNGT in our study was predicated on a number of factors, including the research question, the study objectives, the necessity for consensus, and the practical considerations and constraints associated with the study, such as the time commitment of participants and the geographical scale of Brazil. Given the challenges of running in-person events during the COVID-19 pandemic [32] and the desire to include participants from different Brazilian regions, a careful adaptation of the traditional technique was used to allow for its application in an online format [33]. Supplementary file (Additional file 1).

### Participants

Nurses working professionally in Brazil in different contexts were invited, including members of hospital IPC committees, public health organizations, academics and researchers, frontline nurses from general hospitals, primary health care (PHC), outpatient settings, and professional normative bodies such as the Regional Nursing Council.

To ensure a proportional distribution of guests according to their work context among the five regions of Brazil, a proportion matrix was constructed according to the percentage distribution of health facilities and university hospitals in the country. The proportion matrix included four categories: PHC and outpatient clinics, general hospitals, universities, and the “other” category included health surveillance agencies, professional normative bodies, and IPC professional associations. To assist in the construction of the matrix, a search was carried out in the database of the National Register of Health Establishments [34] and the Ministry of Education [35] to determine the percentage of health facilities and universities distributed in the five regions of Brazil. Details of the distribution of healthcare facilities and the proposed matrix of participants are available in the supplementary file (Additional file 2).

To ensure equitable participation and a more efficient discussion, it is recommended that the number of NGT participants range between seven and 10 guests [25]. The number of guests was carefully planned with the expectation that approximately 30 individuals would participate in the consensus group. By utilizing the recommended group size, three groups, each consisting of a maximum of ten professionals, were envisioned. Each group was balanced regarding region, job position, and health settings to minimize heterogeneity.

### Ethical approval

The study received ethical approval from the Research Ethics Committee of the School of Nursing of University of São Paulo, with approval number 5.381.334. All participants signed an informed consent form before taking part in the virtual workshop.

### Recruitment of participants

Initially, the recruitment of participants involved inviting nurses nominated by the research executive team. Subsequently, snowball sampling was employed to identify potential participants. The participants were then asked to identify others who could fulfill the research inclusion criteria. Those individuals who consented to participate in the research were notified via email with the invitation letter, consent form, and additional details regarding the virtual workshop, including the program, explanations about the mNGT, and a list of publications on the subject.

### Data collection

Data were collected during a virtual workshop called “*The Role of Nursing in Antimicrobial Stewardship Programs*” using the Google Meet^®^ platform with an upgrade to Google Workspace^®^. The workshop consisted of three four-hour sessions (12 hours in total) over three days, with a two-week break between each day, and took place between May and June 2022. The aim of the workshop was to identify and prioritize knowledge gaps concerning nurses' engagement in ASPs in the Brazilian context. All the sessions were recorded (audio and video), and field notes were taken. The authors of this paper actively participated in the workshop from its initial planning stage, serving as lecturers, hosts, facilitators, and supporters during the mNGT and as managers of information and communication technology (ICT).

On the first day of the workshop, the participants were provided with information about AMR, the actions taken to address the problem, and the potential nurses’ role in ASPs. The agenda included short lectures and moments dedicated to questions and clarifications on the subject. The meeting also discussed ASP in the Brazilian context and the ongoing research on the involvement of nurses in ASPs.

**Fig. 1:**
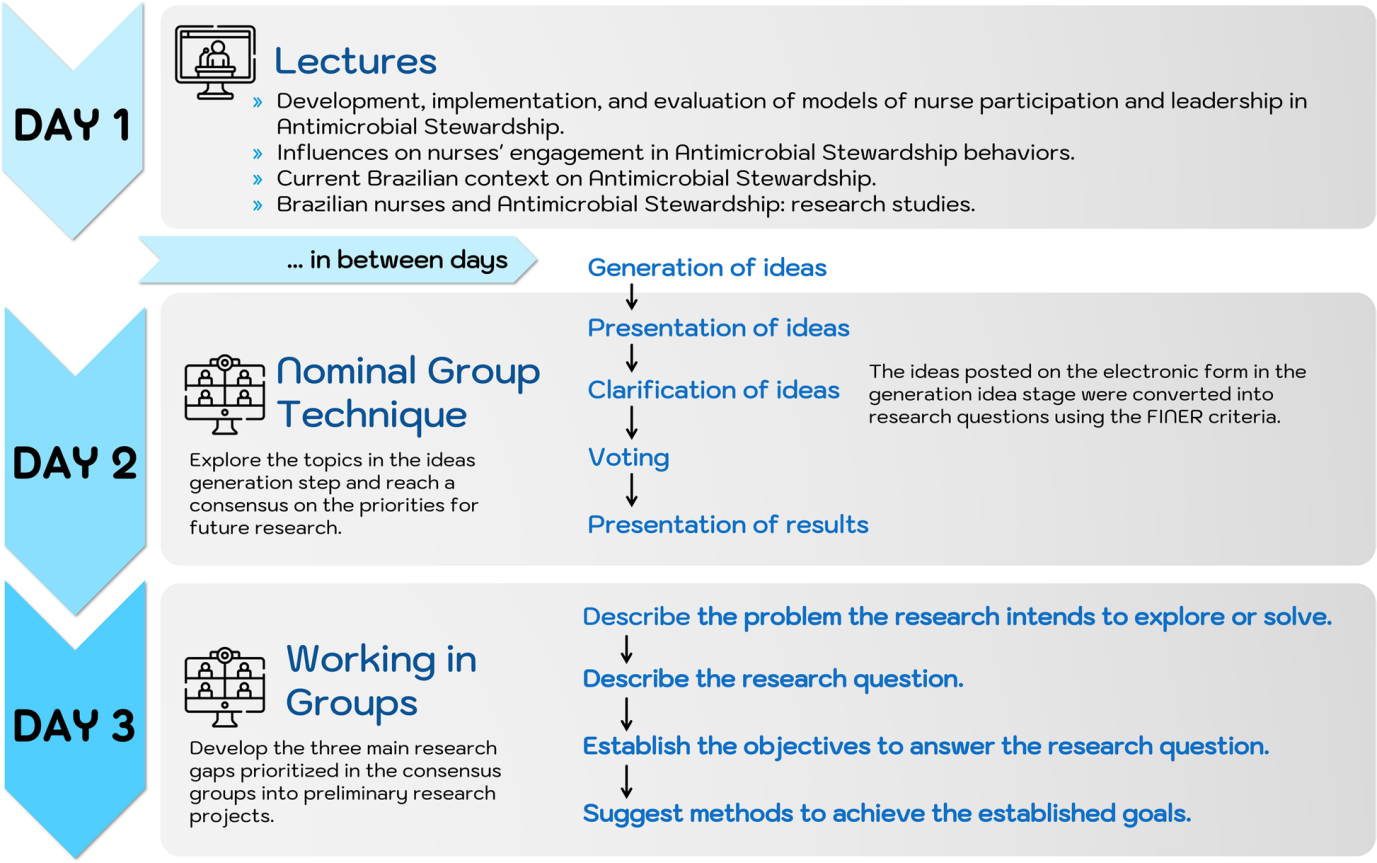
Planning the online workshop

After day 1, the participants received, via email, a link to a digital form containing the guiding question, “*What do you consider to be an important research gap to explore regarding the role of nursing in antimicrobial stewardship?*”. In the form, participants were invited to submit up to three ideas on what they considered to be important research gaps to explore. The electronic form contributed to the idea generation stage of the mNGT. Considering their own views and work experience, the participants were instructed to answer the form with up to three ideas considered research gaps to be explored in future investigations (Fig. 1).

After a two-week interval, the second day of the four-hour workshop was held. Day 2 aimed to explore the preliminary ideas proposed by the participants on the first day and to reach a consensus on the priorities for future research. All the participants were received in the main virtual room, called the “*plenary*”. The host explained the importance of the research question and used the FINER criteria—feasible, interesting, novel, ethical, and relevant—to ensure that appropriate research questions could be formulated [36, 37]. The participants were advised that ideas entered into the electronic form during the idea generation stage should be converted into research questions via the FINER criteria. To clarify and discuss the ideas previously identified, the participants were allocated into three small groups (A, B, and C), with up to 10 participants in each group. The division of the groups considered the excessive time and dispersion caused by tiredness that could be generated by discussing all the ideas with all the participants. Therefore, the discussions took place independently in each thematic room, limiting themselves to the ideas generated by their participants. Each group was made up of participants according to the proportion matrix and assigned a facilitator and a supporter.

The purpose of the presentation and clarification phase was to clarify the ideas entered in the electronic form. The ideas were then converted into research questions. If the ideas were similar and the group agreed, the facilitator adjusted such as combining them or editing or deleting repetitions. The voting round took place in two stages via the SurveyMonkey^®^ virtual platform. First, in the breakout room, participants voted anonymously to choose up to three relevant research questions for future investigations according to their point of view. The second round of voting took place in the plenary room, where each participant and the facilitator could vote individually on a single most relevant research question. Two rounds of voting were chosen so that all participants could discuss and vote on the most relevant research questions in each breakout room. At the end of the process, the participants prioritized the research questions democratically.

After a three-week break, day 3 was held. The aim was to develop the three main research gaps prioritized in the consensus groups during day 2 into preliminary research projects. The methodology used was based on a group discussion following a previously prepared roadmap. The participants were divided into three groups, where each group worked on one of the three prioritized research questions. At the end, the participants presented their projects ideas in the plenary room.

### Data analysis

The results of the mNGT and the demographic data of the participants are presented via descriptive statistics (frequencies, means, standard deviations, and ranges). Additionally, a content analysis was performed on the research questions after the idea’s clarification stage to characterize and identify common topics categories in the proposed research priorities [38, 39]. Two researchers carried out the analysis independently in the categorization phase (AMSF and VCLG). Conflicting categorizations were discussed and agreed upon. A third researcher (MCP) then assessed the relevance of the categorization. Whenever applicable, the participants' quotations are presented in this manuscript in order to illustrate the results of the discussions held in the subgroups.

## Results

During the planning phase, 69 nurses were invited, but only 26 (37.7%) attended. Of the 26 participants, not all were able to attend all three sessions in their entirety; on the third day, only 20 participants were present. The participants were predominantly female (*n *= 22, 84.6%) and aged between 30 and 63 years (mean 43.5 years, SD 8.2). Nurses from all five Brazilian regions took part, covering ten of the twenty-six states and the Federal District. However, compared to the proportion matrix developed in the planning phase, the participation of Northeast and South Brazilian regions was lower than expected. With respect to the practice areas, PHC presented the smallest number of participants. They had professional experience between eight and 39 years (mean 20.6 years, SD 8.1), with the most prevalent work in hospital environments (*n *=11 nurses, 42.3%), followed by professionals working in academic environments developing research (*n *= 6, 23.1%). Most of the participants (*n *= 20 nurses, 73.1%) had no experience with the NGT method (Table 1).

**Table 1:** Demographics of workshop participants

**Participants characteristics**	**Distribution**
Age, mean (SD), range years	43.5 (8.4) 30-63
Professional experience, mean (SD), range years	20.5 (8.2) 8-39
**Gender, *n*** **(%)**	
* Female*	22 (84.6)
* Male*	4 (15.4)
**Professional expertise,** ***n*** **(%)**	
* Hospital*	11 (42.3)
* Academic*	6 (23.1)
* Primary Health Care (PHC) and Outpatient*	4 (15.4)
* Management*	3 (11.5)
* Professional Association*	1 (3.8)
* Nursing Council*	1 (3.8)
**Brazilian Region,** ***n*** **(%)**	
* Southeast*	12 (46.2)
* Central-West*	4 (15.4)
* Northeast*	4 (15.4)
* North*	3 (11.5)
* South*	3 (11.5)
**Prior experience with NGT,** ***n*** **(%)**	
* No*	23 (88.5)
* Yes*	3 (11.5)

**Fig. 2:**
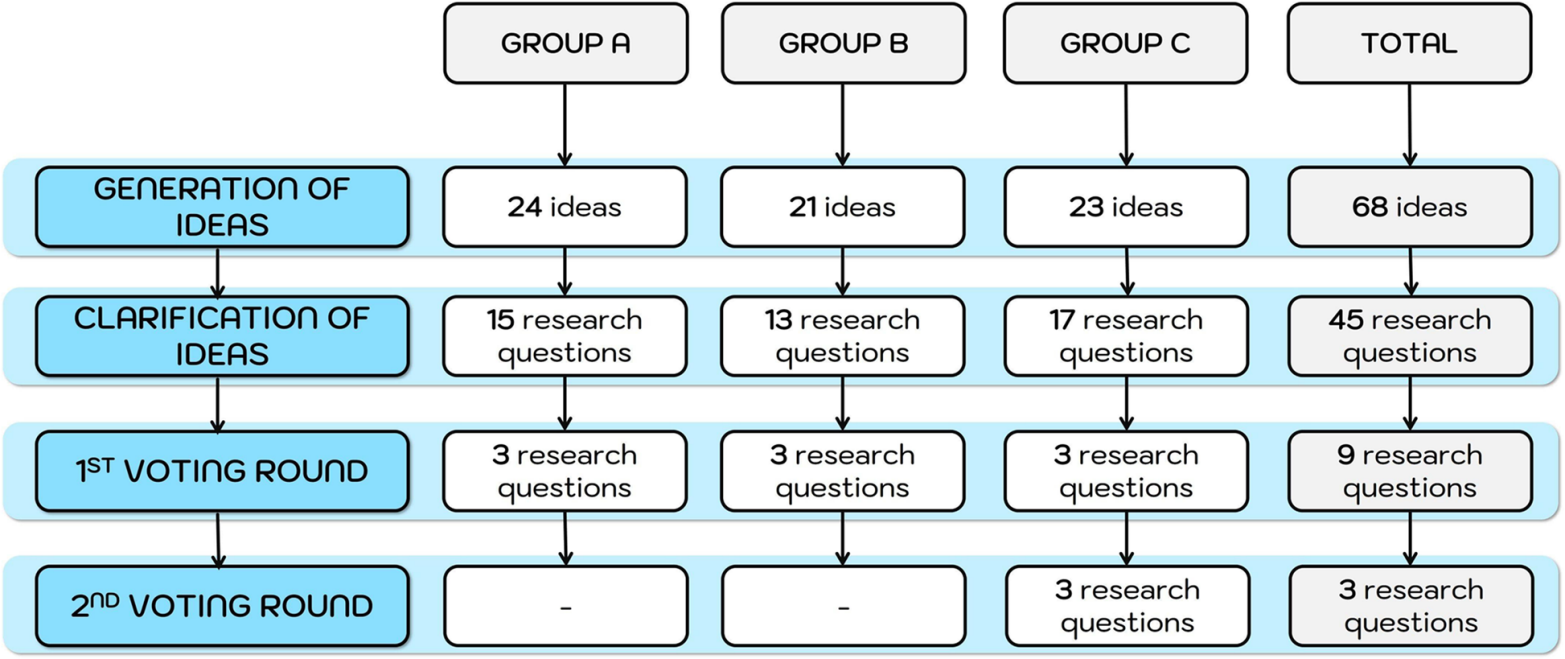
Development of the number of ideas during the mNGT stages

The participants generated 68 preliminary ideas for discussion. In order to ensure the feasibility of the proposed research question, we defined a limited number of preliminary ideas to avoid the generation of a non-operational list. In the subsequent clarification phase, the ideas were subjected to further discussion and modification whenever applicable, either through consolidation or outright rejection, contingent on the group's assessment of their intrinsic value. Therefore, the initial ideas were transformed into 45 research questions, representing a reduction of 33.8% of the original proposition from participants. For pragmatic reasons, after the first round of voting, each subgroup was asked to prioritize only the top three research questions to be ranked in the second round of voting, ensuring a focused and streamlined process for selecting the final nine ideas. During the second round of voting, the three most relevant research questions were prioritized by each group. (Fig. 2). Details of the ideas are available in the supplementary file (Additional file 3).

The research questions that garnered the majority of votes in the second round of voting focused on the following subjects: i) the roles and competencies of nurses in ASPs; ii) the planning and execution of nurses' activities in ASPs; and iii) the utilization of digital technologies to aid nurses in ASPs (Table 2).

**Table 2:** Result of the second round of voting (plenary)

**Research Questions**	***n* (%)**
What are nurses' competences in regard to managing antimicrobials?	8 (26.7)
How should nurses plan and implement their antimicrobial stewardship activities?	6 (20.0)
How can digital technologies help nurses in ASPs?	5 (16.7)
What knowledge do nurses have about the factors that interfere with ASPs?	4 (13.3)
What teaching strategies can be used to educate nursing technicians, undergraduates, and postgraduate students about ASPs?	3 (10.0)
Do professional nurses know their role in ASP?	1 (3.3)
Has providing undergraduate nursing students with knowledge about antimicrobial stewardship resulted in clinical practice?	1 (3.3)
What are the duties and responsibilities of the care nurse in an ASP?	0 (0.0)
What is the level of knowledge that undergraduate nursing students possessing regarding antimicrobial stewardship?	0 (0.0)
**Total**	**30 (100.00)**

Although it was not the aim of this study, it was noted that during the clarification stage, participants had difficulty converting the preliminary ideas generated into relevant research questions. This was observed when the ideas were portrayed in sentences containing only the main theme: “*Nursing training needed*” [Group A], "*Ongoing education with professionals*" [Group B], or in long texts. The facilitators provided opportunities for groups to overcome this challenge through discussion and understanding.

After the workshop, a content analysis of the research questions that emerged during the clarification phase was performed to explore the results of the discussion. Group work recordings and field notes were used. The results of this analysis are shown in Table 3.

**Table 3:** Results of the content analysis of the ideas by the participants

**Topics**	***n* (%)**
(1) Nurses’ knowledge of AMR/ASP and nurses’ education in ASPs	16 (35.6)
(2) Outcomes of nurses’ participation in the ASPs	7 (15.6)
(3) Nurses’ attributions and practice in ASPs	6 (13.3)
(4) Implementation of nurse’s participation in ASPs	4 (8.9)
(5) Barriers for nurses' engagement in ASPs	4 (8.9)
(6) Nurses' autonomy and leadership related to ASPs	3 (6.7)
(7) Nurses’ competencies for ASPs	2 (4.4)
(8) Public policies related to the nurse’s role in ASPs	2 (4.4)
(9) Technological innovations to promote the nurse’s engagement in ASPs	1 (2.2)
**Total**	**45 (100.0)**

The most prevalent topics related to nurses’ learning needs, as well as their motivation, are related to AMR and the functioning of the ASP. This aspect of education was highlighted in the discussions.

*“... The fact that a nurse doesn't feel a part of antimicrobial stewardship, which makes them even less interested in knowing about the use of these drugs…” *[Group C participant].

The participants expressed concern regarding the curriculum of undergraduate courses currently utilized in Brazil, which allocates a limited number of hours to subjects such as microbiology, parasitology, pharmacology, and health and disease processes.

*“I believe that what is currently taught in undergraduate programmes is not sufficient for nurses to have a background that will enable them to engage with the ASP.” *[Group A participant].

*“… Basic subjects need to be strengthened in undergraduate programs ... We've observed that undergraduate courses reduce the number of hours spent on basic subjects such as pharmacology and microbiology, restricting them to the minimum number of hours defined in the curriculum. The teaching of microbiology and pharmacology is left to the eighth level. It's the bare minimum…” *[Group B participant].

According to the participants' perspective, the inadequate provision about of AMR to these subjects may hinder nurses' comprehension of AMR and its implications for their clinical practice. The topic of nurses' prior knowledge regarding AMR, its objectives, and the functioning of ASPs was addressed within the realm of professionals’ expertise. According to the participants, it is important to carry out research to measure this knowledge to structure a relevant educational program.

*“... I think a priority is to find out what nurses know about AMR and basic issues. If we know this information, we can plan studies to strengthen the curriculum matrix or courses aimed at the gaps identified”. *[Group B participant].

With respect to the outcomes of nurses' participation in ASPs, the participants suggested research on how to evaluate the efficacy of nurses' participation in interdisciplinary rounds, influence on antimicrobial prescribing, and proper collection of cultures.

*“We haven't yet looked at how nurses are doing in terms of spotting infections early on, in the context of antimicrobial stewardship programs. It's important that nurses know how to spot infections, but it's not enough to just know. They have to put their knowledge into practice.” *[Group C participant].

*“… there has been no research into the importance of nurses participating in rounds with the multidisciplinary team when decisions are being made about antimicrobial stewardship. And this has a lot to do with basic knowledge about antimicrobials... professionals are often not empowered because they don't have this knowledge. They don't remember, they don't review, right? And then they just accept what's said…” *[Group C participant].

*“It would be interesting to clarify the role of nurses during the collection of cultures in the technique and all the care taken with the sample*.” [Group C participant].

The participants emphasized the importance of conducting research into the working process and the roles of nurses in practice:* “What are the general and specific attributes of nurses in ASPs?”, “Do nurses know their role in the ASP?” *[Group A];* “What is the nurse's role in the early detection of infection in the ASP?” *[Group C].

The topic of implementation discussed aspects of implementing nurses' participation in the ASPs in different work contexts. The participants also addressed the barriers that prevent nurses from participating in ASPs, as well as their perceptions of these barriers: *"What factors hinder nurses’ participation in the ASP?"* [Group A], *“What factors hinder the nurse's work in controlling antimicrobial stewardship?”*, *“What is the nurse's perception of the factors that interfere with their participation in the ASP?”* [Group B]. However, during the mNGT, no topics related to the investigation of facilitators or accelerators were spontaneously identified.

With respect to nurses' autonomy, we addressed the perceptions of the nursing team and the multidisciplinary team about the nurses' performance in the ASPs. The participants expressed the importance of researching nurses' competences in the ASPs, such as their skills, attitudes, values, and capabilities.

*… it is very important to conduct research that addresses competencies, what their role would be in practice and what attitudes would be expected of nurses in antimicrobial stewardship programs…* [Group A participant].

Only one participant presented a research proposal that inquired about the potential benefits of digital tools for nurses in ASPs. Curiously, this research question was placed third in the final priority list. Notably, the three priorities obtained in the mNGT emerged from group C (Fig. 2).

## Discussion

Despite the substantial number of studies conducted in Europe, the United States, and Australia that examine the significance of nursing in ASPs [11, 17, 18, 21, 23, 40–43], there is a lack of knowledge regarding the role of nurses in these programs in Brazil. To our knowledge, there is no similar study on research priorities for nurses’ role in ASPs in low- and middle-income countries. A recent review conducted in Brazil [20] that examined the available evidence regarding nursing strategies in ASPs in hospitals revealed that, despite the recent involvement of Brazilian nurses in ASPs, there is a dearth of Brazilian studies published on the subject. To address this challenge, the objective of this investigation was to identify and categorize the priority areas for future research from the nurses' perspective via the mNGT.

Despite claims that nurses should be engaged with ASPs, in many countries, such as Brazil, nurses remain disconnected from this activity. A national evaluation survey in Brazilian adult intensive care units revealed that although 667 out of the 863 (77.29%) hospitals reported that they had the human resources needed to implement ASPs, only 497 (57.6%) institutions reported that there were nurses on the ASPs management teams [44].

The field of nursing research is characterized by a dynamic evolution and maturation process, whereby the findings of previous studies contribute to the expansion of knowledge in a given area [45]. In light of these considerations, the preliminary ideas that emerged during the generation ideas stage indicated that the role of nursing in addressing AMR and, more specifically, its involvement in ASPs remain under-researched areas in Brazil. This may be indicative of the fact that the level of maturity currently reached within the national research context remains incipient. During the group discussions, the preliminary suggested ideas were elucidated and research questions emerged, including the implementation of interventions, the use of technologies, and the evaluation of the effectiveness of ASPs with the participation of nurses. Although not all the time recognized, nurses play a central role in antimicrobial management, patient education, and infection prevention [18, 20]. Therefore, understanding and optimizing the role of nursing in ASPs is essential for effective healthcare delivery.

One of the priorities pointed out by the participants was mapping nurses' attributions in the ASPs. Future research should focus on the description of roles and competencies, the general and specific attributes of front-line nurses in ASPs and the early detection of infections. Defining roles and responsibilities is an essential step in behavior change to make the work environment more fluid and efficient. Studies have indicated that the lack of definition of roles is a considerable barrier to the implementation of ASPs, as it leads nurses further from their expected duties, generates interprofessional dissonance, lacks recognition, reduces productivity, and causes professional dissatisfaction [23, 46, 47].

The participants indicated that although nurses already carry out activities related to the ASP, neither they nor other professionals consider these actions to be important for them to become part of the decision-making process. These findings were similar to those of the reviews published by Gotterson et al. [23] and Bos et al. [18], which both emphasize that nurses' contributions, despite being grounded in clinical practice, are not yet acknowledged as a component of the ASP.

The participants suggested research questions that reinforce the call for research into how to implement nurses' participation in ASPs, including identifying barriers to this participation. Although the Brazilian Health Regulatory Agency (Anvisa) has updated the national guidelines for the development of ASPs in healthcare facilities, strategies for involving nurses have not yet been addressed [48]. One study recommended the use of tools such as the Plan-Do-Study-Act (PDSA) framework to implement the role of nurses in conjunction with a meticulous assessment of the organizational culture and obstacles, available resources, and workflows [17]. A literature review by Gotterson et al., [23] highlighted the advantages of applying behaviour change theory as a strategy to encourage nurse engagement. However, up to our knowledge, none of these alternatives have been applied to the Brazilian scenario to assess the viability of these proposals.

Digital technologies to support the nurse’s contribution in the ASPs were also discussed during the mNGT. According to participants, by harnessing the capabilities of digital technologies, nurses can strengthen their contributions to antimicrobial stewardship efforts. These tools can empower nurses with the information, support, and resources needed to optimize antimicrobial use and promote responsible prescribing practices. Research has explored technological innovations in nursing education with significant results [49]. A study described how technology, grouped into a set of ICT tools, can support nurses and doctors in making decisions about the prescription and administration of antimicrobials [50]. The authors delineated the manner in which artificial intelligence has been employed to facilitate the generation of alerts and notifications pertaining to patients' clinical status, and its role in aiding the conduct of clinical audits of ASP results [50] Nonetheless, the utilization of ICT tools to aid nurses in ASPs in Brazil still requires exploration, as it has the potential to increase the knowledge and engagement of these professionals.

### Strengthens and limitations of the study

The limitations of this research are related to the limited number of participants, which is inherent to the NGT itself and does not allow more guests to give their opinions to each group. However, an attempt was made to minimize this limitation through proportional distribution in terms of regional representation and the participants' institutions of origin. The number of participants was representative but not sufficient to allow an exhaustive approach to the subject. Another limitation was that not all the participants were able to discuss and vote on all 45 ideas since they were organized into themed rooms, but considering the operational issue, this was a pragmatic decision from the researchers. The use of mNGT in the virtual environment itself could be a limitation. However, it was conducted entirely online using free ICT resources, eliminating travel or accommodation costs [33]. Participant numbers and geographic location posed no limitations. The workshop proceeded smoothly, with no issues related to internet connectivity or virtual room navigation, and all voting rounds were completed as planned. The agenda was followed according to the schedule.

Finally, the presentations offered to participants might have influenced their decisions about research priorities. However, as this is an incipient topic in Brazil, we believe that providing clear, relevant, and accurate information led to more discussions and better results, as well as collaboration towards a collective contribution from the participants in a fair and balanced way.

The development of this study contributed to the direction of strategies proposed by the Brazilian nurses who work to address AMR. The creation of a network of Brazilian nurses dedicated to addressing antimicrobial resistance represents a significant step forward, fostering collaboration and laying the groundwork for future research in this critical area. The research questions that emerged during the mNGT in the form of research questions have been incorporated into the research project which are currently under development.

## Conclusion

This study identified significant research gaps regarding the role of nurses in ASPs within the Brazilian context. These gaps include the need for further exploration into nurses' competencies, the implementation of ASP activities, and the utilization of digital technologies to support nursing involvement. These findings highlight the necessity for the development of bespoke research initiatives with the objective of enhancing the contribution of nurses to ASPs, particularly in low- and middle-income countries.

## Supplementary Information

Supplementary Material 1.

Supplementary Material 2.

Supplementary Material 3.

## Data Availability

All the data generated or analysed during this study are included in this published article [and its supplementary additional files]. Original data are available from the corresponding author upon reasonable request.
