# Supplementary material for: Research priorities for antimicrobial stewardship nurses in a middle-income country: a nominal group technique study
Source: BMC Nurs. 2024 Dec 2;23:870. doi: 10.1186/s12912-024-02504-9 (PMC11610058; doi:10.1186/s12912-024-02504-9)
Supplement: Supplementary file 1 — Supplementary Material 1. [file 12912_2024_2504_MOESM1_ESM.docx]

**Additional file 1.** Comparison of the original NGT and modified NGT steps and brief descriptions.

| *Stages* | *NGT Traditional* ^(24)^ | *Modified NGT (mNGT)* ^(28)^ |
| --- | --- | --- |
| *Generation of ideas* | The facilitator guides the participants to write down their ideas in short phases or statements according to the guiding question. | The organizing team provided the participants with an idea collection form using the electronic form application containing the guiding question. To maintain the confidentiality of the participants, each participant was asked to identify themselves using a nickname. Each participant could enter up to three ideas that they considered to be research gaps. |
| *Presentation of ideas* | Each participant presents, without discussion, one of the ideas from their list, in presentation rounds. The ideas are recorded by the supporter for all to see. | The ideas were organized using Microsoft Excel 2019 spreadsheet software and shared via Google Drive.  Participants had ample time to present their ideas and discuss them among themselves. |
| *Clarification of ideas* | Participants express the relative importance of each idea. If there is duplication, ideas can be combined with the agreement of the group | All participants had the opportunity to express their opinions and clarify their doubts about the meaning and interpretation of the ideas previously exposed. If there were any duplicate ideas, they could be combined or excluded based on the collective consensus. |
| *Round Voting* | Participants vote privately to prioritize the ideas, using criteria created by the facilitator.  The facilitator will add the assigned scores.  The results are then discussed in the group and recorded by the supporter, with the rank order of the issues and concerns identified | The facilitator asked the participants to vote on the most relevant research question using the SurveyMonkey platform. Participants received voting links for their respective survey questions via Google Meet^®^ chat and were instructed to use their cell phones to access the survey link and vote individually and anonymously. When the voting stage was completed, the application provided a ranking of the most voted ideas. The result was shared in full of the participants. |
