# Supplementary material for: Research priorities for antimicrobial stewardship nurses in a middle-income country: a nominal group technique study
Source: BMC Nurs. 2024 Dec 2;23:870. doi: 10.1186/s12912-024-02504-9 (PMC11610058; doi:10.1186/s12912-024-02504-9)
Supplement: Supplementary file 2 — Supplementary Material 2. [file 12912_2024_2504_MOESM2_ESM.docx]

**Additional file 2** Details of the distribution of healthcare facilities, and the proposed matrix of participant’s

Figure 1. Percentage distribution of health facilities by region in Brazil, 2022 (%).

Primary Health Care: 49.577

General Hospitals: 6.513

University Hospitals: 41

Source:

National Register of Health Establishments <http://cnes2.datasus.gov.br/> - Extraction date: 19/03/2022.

Ministry of Education <http://portal.mec.gov.br/hospitais-universitarios?id=13808> – Extraction date: 19/03/2022.

Table 2. Proportion matrix with distribution of participants according to type of health facility and regions of Brazil, 2022 (n).

|  | ***Central-West*** | | ***Northeast*** | | ***North*** | | ***Southeast*** | | ***South*** | | ***Total*** | |
| --- | --- | --- | --- | --- | --- | --- | --- | --- | --- | --- | --- | --- |
|  | ***Planned*** | ***Acquired*** | ***Planned*** | ***Acquired*** | ***Planned*** | ***Acquired*** | ***Planned*** | ***Acquired*** | ***Planned*** | ***Acquired*** | ***Planned*** | ***Acquired*** |
| *Outpatient* | 1 | 1 | 3 | 0 | 1 | 1 | 3 | 2 | 1 | 0 | 9 | 4 |
| *Hospitals* | 1 | 1 | 2 | 2 | 1 | 1 | 3 | 6 | 1 | 1 | 8 | 11 |
| *Academics* | 1 | 1 | 3 | 2 | 1 | 0 | 3 | 2 | 1 | 1 | 9 | 6 |
| *Other** | 1 | 1 | 1 | 0 | 1 | 1 | 1 | 2 | 1 | 1 | 5 | 5 |
| Total | 4 | 4 | 9 | 4 | 4 | 3 | 10 | 12 | 4 | 3 | 31 | 26 |

*Healthcare surveillance agencies, professional councils, nursing management, professional associations experts in IPC.
