# Supplementary material for: Research priorities for antimicrobial stewardship nurses in a middle-income country: a nominal group technique study
Source: BMC Nurs. 2024 Dec 2;23:870. doi: 10.1186/s12912-024-02504-9 (PMC11610058; doi:10.1186/s12912-024-02504-9)
Supplement: Supplementary file 3 — Supplementary Material 3. [file 12912_2024_2504_MOESM3_ESM.docx]

**Additional file 3** Result of the first round of voting

Table 3. Result of the first round of voting – Group A.

| ***Research Questions*** | ***Number of votes (n)*** | ***%*** |
| --- | --- | --- |
| Are there continuing education proposals (in-service education) for nursing professionals about ASPs in health institutions? | 1 | 4.8 |
| How does the front line and management team see the nurse in ASPs? | 1 | 4.8 |
| Is there an approach to ASPs and microbial resistance in technical nursing courses? | 0 | 0.0 |
| What teaching strategies can be used in the training of nursing technicians, undergraduate and graduate students on ASPs?* | 4 | 19.0 |
| How does the workload influence the nurse's participation in ASPs? | 2 | 9.5 |
| How to implement an antimicrobial stewardship program in Emergency Department units? | 1 | 4.8 |
| Do nurses know their role in ASPs? * | 3 | 14.3 |
| What are the factors that interfere with the participation of the professional Nurse in ASPs? | 1 | 4.8 |
| Does the professional Nurse have autonomy/voice in the ASPs? | 0 | 0.0 |
| Is time out an effective strategy for nurses in ASPs? | 0 | 0.0 |
| What is the nurse's necessary knowledge in the transition of the antimicrobial administration route? | 0 | 0.0 |
| What do nurses need to know to act effectively and contribute to an ASPs? | 1 | 4.8 |
| What are the general and specific attributions of the IPC nurse in an ASPs? | 3 | 14.3 |
| What are the general and specific attributions of the assistant nurse in an ASPs?* | 3 | 14.3 |
| How to enable the active and autonomous participation of nurses in ASPs? | 1 | 4.8 |
| Total | 21 | 100.00 |

Table 4. Result of the first round of voting – Group B.

| ***Research Questions*** | ***Number of votes (n)*** | ***%*** |
| --- | --- | --- |
| What is the perception of primary health care professionals in relation to their role in ASP? | 0 | 0.0 |
| Does the practice of continuous review of antimicrobial prescriptions by nurses decrease the consumption of these drugs? | 1 | 5.6 |
| What is the knowledge of undergraduate nursing students about antimicrobial management? ***** | 4 | 22.2 |
| How to empower nurses on the management of antimicrobial use and AMR identification? | 1 | 5.6 |
| What are the public policies aimed at the nurse's leadership in the management of antimicrobial use? | 0 | 0.0 |
| What are the permanent and continuous education programmes in the training of professionals in ASPs? | 2 | 11.1 |
| What are the programmes for education of the patient in ASPs? | 2 | 11.1 |
| What are the nurses' knowledge factors that interfere in ASPs?***** | 3 | 16.7 |
| What is the effectiveness of nurses' participation in a multi-professional round? | 0 | 0.0 |
| What factors make it difficult for nurses to control antimicrobial management? | 1 | 5.6 |
| Has instrumentalizing nursing students during graduation with knowledge about the control of antimicrobial management resulted in the clinical practice of these professionals?***** | 3 | 12.5 |
| Is the prescriber's conduct changed after the continuous review of the prescription performed by the nurses? | 0 | 0.0 |
| Does the ASPs nurses influence the front-line nurses to adopt practices to control the use of antimicrobials? | 1 | 5.6 |
| Total | 18 | 100.0 |

Table 5. Result of the first round of voting – Group C.

| ***Research Questions*** | ***Number of votes (n)*** | ***%*** |
| --- | --- | --- |
| What is the nurse's influence in the management of antimicrobials? | 1 | 3.3 |
| How should the planning and implementation of nurses' activities in antimicrobial management occur?***^§^** | 4 | 13.3 |
| What are the attributions of the nurse in ASPs? | 3 | 10.0 |
| What is the nurse's knowledge of antimicrobials? | 1 | 3.3 |
| What is the primary health care nurse's knowledge about antimicrobials and their relationship with antimicrobial resistance? | 2 | 6.6 |
| Is the issue of antimicrobial management addressed in the undergraduate nursing curriculum? | 0 | 0.0 |
| Is the topic of antimicrobial management addressed in postgraduate courses in nursing? | 2 | 6.6 |
| What is the importance of nurses in collecting cultures within the ASPs? | 0 | 0.0 |
| What is the nurse's role in the early detection of infection in the context of ASPs? | 0 | 0.0 |
| What is the nurses' knowledge about antimicrobial use? | 3 | 10.0 |
| What are the nurses' competences regarding the management of antimicrobials?***^§^** | 5 | 16.7 |
| What are the necessary elements for the elaboration of public policies in ASPs aimed at ASPs nurses? | 3 | 10.0 |
| What are the competencies of the Primary Health Care nurse in the ASPs? | 0 | 0.0 |
| What is the level of knowledge of nurses about the mechanism of action of antimicrobials? | 1 | 3.3 |
| What is the level of knowledge of nurses about antimicrobials and the importance of respecting the prescribed times and dosages? | 0 | 0.0 |
| What is the nurse's role in guiding patients in relation to the appropriate follow-up of antimicrobial prescriptions? | 1 | 3.3 |
| How can digital technologies help nurses in ASP?***^§^** | 4 | 13.3 |
| Total | 30 | 100.0 |
